# Role of protein degradation systems in colorectal cancer

**DOI:** 10.1038/s41420-023-01781-8

**Published:** 2024-03-14

**Authors:** Zihan Cui, Mingqi Cong, Shengjie Yin, Yuqi Li, Yuguang Ye, Xi Liu, Jing Tang

**Affiliations:** 1 Department of Pathology, Harbin Medical University, Harbin, 150081 China; 2 Department of Oncology, Chifeng City Hospital, Chifeng, 024000 China; 3 Department of Gynecology, Harbin Medical University Cancer Hospital, Harbin, 150081 China; 4 Cardiovascular Center, Inner Mongolia People’s Hospital, Hohhot, Inner Mongolia, 010017 China

**Keywords:** Colorectal cancer, Cell death

## Abstract

Protein degradation is essential for maintaining protein homeostasis. The ubiquitin‒proteasome system (UPS) and autophagy–lysosome system are the two primary pathways responsible for protein degradation and directly related to cell survival. In malignant tumors, the UPS plays a critical role in managing the excessive protein load caused by cancer cells hyperproliferation. In this review, we provide a comprehensive overview of the dual roles played by the UPS and autolysosome system in colorectal cancer (CRC), elucidating their impact on the initiation and progression of this disease while also highlighting their compensatory relationship. Simultaneously targeting both protein degradation pathways offers new promise for enhancing treatment efficacy against CRC. Additionally, apoptosis is closely linked to ubiquitination and autophagy, and caspases degrade proteins. A thorough comprehension of the interplay between various protein degradation pathways is highly important for clarifying the mechanism underlying the onset and progression of CRC.

## FACT

- The intricate interplay between the ubiquitin-proteasome system (UPS) and autophagy-lysosomal system in maintaining protein homeostasis raises questions regarding the regulatory mechanisms and crosstalk between these pathways.
- Understanding the specific factors that determine the preference for UPS or autophagy-lysosomal system-mediated protein degradation in different cellular contexts, including colorectal cancer (CRC), remains an active area of investigation.
- The extent to which the dysregulation of UPS and autophagy influences the development and progression of CRC, including its impact on tumor initiation, growth, and metastasis, requires further exploration.
- Investigating the compensatory relationship between the UPS and autolysosome system in CRC may reveal novel therapeutic strategies that simultaneously target both pathways, aiming to improve treatment efficacy and overcome drug resistance.

## Open questions

- What are the specific molecular mechanisms that regulate the balance between the UPS and autophagy-lysosomal system in maintaining protein homeostasis in normal cells, and how is this balance disrupted in malignant tumors?
- Are there specific factors or signaling pathways that determine the preferential utilization of either the UPS or autophagy-lysosomal system for protein degradation in CRC?
- Can targeting both the UPS and autophagy-lysosomal system simultaneously effectively overcome the adaptive responses and resistance mechanisms that cancer cells develop against single-pathway inhibition?
- How does the dysregulation of the UPS and autophagy-lysosomal system in CRC contribute to the acquisition of drug resistance and tumor progression?
- What are the potential therapeutic strategies or combination therapies that can be developed to modulate the protein degradation pathways to enhance treatment efficacy and improve patient outcomes in CRC?

## Introduction

Proteins serve as the main functional products of genetic material in both prokaryotic and eukaryotic organisms. Their activity and function dictate numerous aspects of the organism, rendering protein homeostasis a crucial component in maintaining metabolism and combating disease. The level of proteins in the body is dependent not only on their synthesis rate but also on their degradation [1]. Even in healthy cells, proteins can be produced that require rapid degradation due to abnormal gene expression and misfolding. Some proteins that are initially folded correctly can also aggregate due to environmental factors that increase their surface hydrophobicity [2]. Furthermore, when cells are exposed to proteotoxic conditions such as heat shock, oxidative stress, nutrient starvation and metabolic imbalance, damaged proteins can be produced. The ability of cells to fold and degrade proteins becomes limited over time, resulting in the accumulation of abnormal proteins that cannot be removed in time. This eventually leads to the onset of various diseases [3]. Recent research on cancer has revealed that genomic abnormalities in cancer cells promote protein misfolding and the accumulation of toxic proteins. To ensure their survival and growth, cancer cells employ protein degradation mechanisms to counteract the harmful effects of toxic proteins. This makes the protein degradation pathway a potential target for treating cancer [4]. This review delves into the mechanisms underlying protein degradation in colorectal (CRC), with a focus on the two primary systems involved: the ubiquitin‒proteasome system and the autophagy–lysosome system. This article comprehensively reviews the relationship between two protein degradation pathways and CRC. The aim is to establish a more solid theoretical foundation for understanding the occurrence and development of CRC, and potentially identify new treatment prospects.

## The ubiquitin-proteasome system (UPS)

### Introduction to the UPS

The UPS is a cellular mechanism that assumes responsibility for the degradation of abnormally folded or short-lived proteins. It comprises six primary components: ubiquitin, a ubiquitin-activating enzyme (E1), a ubiquitin-conjugating enzyme (E2), a ubiquitin-ligase (E3), the proteasome, and a deubiquitinase (DUB). Specifically, there are two ubiquitin-activating enzymes, over forty E2 ubiquitin-conjugating enzymes and hundreds of E3 ubiquitin ligases that can make up this system [5].

Protein degradation through the UPS involves two distinct steps. The first step is protein ubiquitination, which is executed in three sequential steps by E1 ubiquitin-activating enzymes, E2 ubiquitin-conjugating enzymes and E3 ubiquitin ligases. Initially, the E1 ubiquitin-activating enzyme activates the ubiquitin molecule and transfers it to the E2 ubiquitin-conjugating enzyme using ATP energy. Subsequently, the E3 ubiquitin ligase identifies the target protein and facilitates the transfer of ubiquitin from the E2 ubiquitin-conjugating enzyme to the protein for ubiquitination [6]. Ubiquitination modifications can be classified into two types: monoubiquitination and polyubiquitination. Monoubiquitination involves the addition of a single ubiquitin molecule to a lysine residue of a substrate protein and plays a role in regulating DNA damage repair. Polyubiquitination, on the other hand, involves the combination of seven lysine residues (K6, K11, K27, K29, K33, K48 and K63) or an N-terminal methionine residue (M1) with another ubiquitin molecule to form a ubiquitin chain, including homotypic chains through a particular lysine on Ub or mixed polyubiquitin chains generated by polymerization through different Ub lysines [7]. For instance, the K6, K11 ubiquitin chain is a type of polyubiquitination. Polyubiquitination can result in different outcomes depending on the type of ubiquitin chain. K48 and K11-linked polyubiquitination are associated with proteasomal degradation, with K48 being the primary signal for targeting 26 S proteasomal substrates. On the other hand, K63-linked polyubiquitination is involved in cellular signal assembly and transduction [8]. After the target protein is polyubiquitinated, it is degraded by the 26 S proteasome in an ATP-dependent manner [9].

**Fig. 1:**
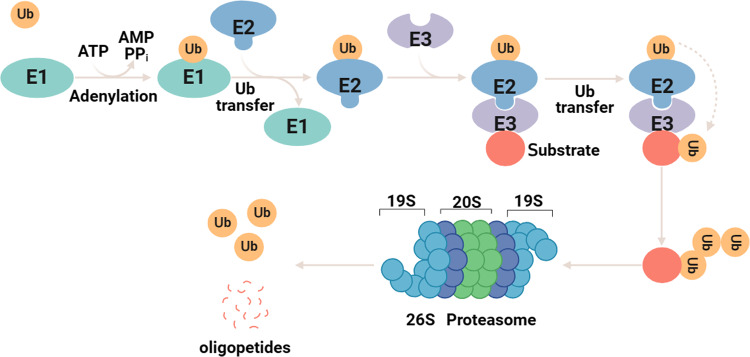
A brief overview of the UPS. The first step is ubiquitin-activating enzyme E1, which consumes ATP to activate ubiquitin. Then, the activated ubiquitin is transferred to the E2 ubiquitin-conjugating enzymes. Finally, the charged E2 enzymes cooperate with one of hundreds of E3 ubiquitin ligases to transfer the activated ubiquitin to a target substrate. The ubiquitinated substrate protein is recognized and deubiquitinated by the 19 S proteasome and subsequently degraded by the 20 S proteasome.

The 26 S proteasome comprises the proteolytic core particle (CP, alternatively named 20 S proteasome) and the regulatory particle (RP, or 19 S proteasome) [10]. The 20 S core complex consists of four concentric rings, each composed of seven subunits, giving rise to a barrel-like structure. The two outer rings of this barrel structure are referred to as the α-ring, consisting of seven α-subunits. The two inner rings are known as the β-ring, made up of seven β-subunits. The binding between the 19 S regulatory complex and the 20 S core complex is facilitated by the α-loop [11]. The 19 S regulatory complex encompasses at least nineteen subunits, encompassing both the base and the lid components. The base is comprised of six distinct homologous AAA+ ATPase subunits, namely regulatory particle triple-A protein 1 (RPT1) to RPT6, alongside three non-ATPase subunits, regulatory particle non-ATPase 1 (RPN1), RPN2 and RPN13 [12]. These ATPase subunits are responsible for the unwinding of substrates and the opening of the α-ring channel opening, both of which are essential steps for the threading of substrates into the 20 S proteasome. RPN1, RPN10, and RPN13 are involved in capturing ubiquitinated proteins [13]. On the other hand, the lid is composed of nine non-ATPase subunits: namely RPN3, RPN5–RPN9, RPN11, RPN12 and RPN15. RPN11 functions as a de-ubiquitylate agent, facilitating the degradation of the captured substrates. Nonetheless, the functionalities of the remaining subunits undisclosed (Fig. 1) [14]. The 19 S RP selectively recognizes proteasomal substrates upon the recognition of polyubiquitin chains, subsequently removing them in conjunction with unwinding and transportation into the 20 S proteasome through the utilization of energy derived from ATP hydrolysis.

The UPS is a crucial component for protein degradation in eukaryotic cells. It is involved in various cellular processes, such as the cell cycle, gene transcription, DNA repair and apoptosis by targeting and degrading specific proteins [5]. Moreover, the UPS plays a significant role in maintaining normal colonic epithelial function. An increasing number of studies have demonstrated that dysregulation of protein degradation caused by ubiquitinated proteasomes can lead to the development of CRC. This paper will extensively discuss the impact of the key enzymes involved in ubiquitination, namely ubiquitin-conjugating enzyme E2, ubiquitin-linked enzyme E3 and DUB, on the progression of CRC.

### The role of dysregulated protein degradation caused by the UPS in CRC progression

#### Role of E1 ubiquitin-activating enzymes

The ubiquitinated proteasomal degradation of proteins in CRC typically entails the orchestrated activities of three enzymes: ubiquitin-activating enzymes (E1s), ubiquitin-conjugating enzymes (E2s), and ubiquitin ligases (E3s). Among these enzymes, the E3 ligase holds the utmost significance within the realm of ubiquitin coupling, as it directly binds to specific substrate proteins. Numerous articles have explored the impact of E3 ligases on the progression of CRC through the mediation of targeted protein degradation. However, there remains a dearth of research pertaining to the specific involvement of E1 ubiquitin-activating enzymes and E2 ubiquitin-conjugating enzymes within the ubiquitin‒proteasome system in relation to CRC [15].

Up to this point in time, a total of eight E1 enzymes have been identified. These include both canonical (UBA1, UBA6, UBA7, SAE, and NAE) and noncanonical (UBA4, UBA5, and ATG7) E1 enzymes. These E1 enzymes initiate more than a dozen post-translational modifications, encompassing ubiquitination, neddylation, SUMOylation, FATylation, ISGylation, URMylation, UFMylation and ATGylation [16]. Among these, UBA1 and UBA6 serve as ubiquitin-activating enzyme, which activate ubiquitin to initiate ubiquitination modifications, leading to protein degradation via the ubiquitinated proteasome pathway. Consequently, they influence the activity, expression, stability, or localization of multiple signaling molecules, ultimately impacting the capacity for biological signaling and the cellular response to stress in the context of cancer [17]. Therefore, targeted manipulation of ubiquitin-activating enzyme E1 holds promise for yielding beneficial anti-tumor effects. Notably, ubiquitylation can indirectly contribute to tumor angiogenesis by promoting the degradation of p53, thereby stabilizing hypoxia-inducible factor 1 (HIF-1). Consequently, therapeutic inhibition of the ubiquitin-activating E1 is expected to decelerate tumor progression [18]. In 2018, a study identified TAK-243 as a highly potent small molecule inhibitor of ubiquitin-activating enzymes. This inhibition mechanism employed by TAK-243 led to the depletion of cellular ubiquitin couplings, resulting in the disruption of signaling events, the induction of proteotoxic stress, and the impairment of cell cycle progression and DNA damage repair pathways. Ultimately, TAK-243 treatment resulted in cancer cell death, which carries profound implications for cancer therapy [19].

#### Effect of the E2 ubiquitin-conjugating enzymes on CRC

The impact of E2 ligases on CRC is twofold and relies on E3 to exert its specificity. For example, the E2 ubiquitin-conjugating enzyme UBE2J1 functions as a suppressor gene, inhibiting the proliferation and metastasis of CRC cells [20]. Mechanistically, UBE2J1-TRIM3 forms an E2-E3 complex that physically interacts with RPS3 and targets K214 residues for ubiquitination and degradation. In CRC, the downregulation of RPS3 induced by UBE2J1 overexpression inhibits the translocation of NF-κB to the nucleus, consequently deactivating the NF-κB signaling pathway. However, the ubiquitin-conjugating enzymes UBE2T, UBE2V1, and UBE2C contribute to the progression of CRC by influencing protein degradation. The ubiquitin-conjugating enzyme UBE2T facilitates the ubiquitination and degradation of p53, thereby promoting CRC [21]. UBE2V1 enhances the metastasis of CRC by facilitating the ubiquitination and degradation of Sirt1 with the assistance of UBC13 (a significant cofactor of UBE2V1), leading to the inhibition of histone H4 lysine 16 acetylation and ultimately suppressing the expression of autophagy genes in CRC [22]. The expression of UBE2C is significantly elevated in CRC. UBE2C ligase, in conjunction with E3, catalyzes the ubiquitination and degradation of mitotic cell cycle proteins A and B1, as well as securin, which promotes cell viability. In the context of CRC, bortezomib stabilizes mitotic cell cycle proteins and impedes cell cycle progression by inhibiting UBE2C [23, 24].

#### Ubiquitin ligase E3 plays a different role in CRC carcinogenesis

The ubiquitination process heavily relies on the ubiquitin ligase E3, which plays a vital role in determining substrate specificity [25]. This ligase is categorized into three families, namely, the RING-finger class E3 ubiquitin ligase family, the homologous to E6AP C-terminus (HECT) family, and the RING-between-RING (RBR) family [26]. The RING-finger E3 ubiquitin ligase family further consists of the RING family, the Cullins family, and the U-box family. The RING-finger E3s act as a bridge to transfer activated ubiquitin directly from E2 to the target protein and do not interact with ubiquitin themselves. The HECT family and RBR family of ubiquitin ligases E3 facilitate the transfer of ubiquitin to substrate proteins in a two-step process. In the first step, ubiquitin is transferred to E3, and in the second step, it is transferred from E3 to the substrate [27]. Numerous studies have demonstrated that E3 ubiquitin ligases play a crucial role in promoting the proliferation, migration and invasion of CRC cells by degrading proteins. For instance, RNF6, a member of the RING family, has been shown to promote cell growth, cell cycle progression and epithelial-to-mesenchymal transition in CRC cells. RNF6 achieves this by ubiquitinating the inhibitor of the β-catenin/TCF4 complex, TLE3, leading to its degradation. This, in turn, inhibits the binding of TLE3 to TCF4, allowing TCF4 to bind to the β-catenin protein, and activate it, thereby promoting the development of CRC [28].

The tripartite motif (TRIM) family is a group of RING-finger E3s. TRIM65 mediates their ubiquitination by binding to ARHGAP35. This leads to the targeting of ARHGAP35 for ubiquitinated proteasomal degradation and an increase in Rho GTPase activity, which promotes CRC metastasis [29]. TRIM47 interacts with SMAD4, which increases SMAD4 ubiquitination and degradation. This results in the upregulation of CCL15 expression, promoting the proliferation and invasion of CRC cells through CCL15-CCR1 signaling [30].

The substrate articulator of the largest E3 ubiquitin ligase SCF complex is the F-box, which plays a crucial role in determining substrate recognition by SCF [31]. In CRC, FBXL6, an F-box protein, is upregulated and highly correlated with poor prognosis in human CRC patients. Li et al. discovered that FBXL6 promotes the degradation of p53 through its ubiquitination at the K48 chain linkage at residues Lys291 and Lys292, which inhibits p53 signaling and thus promotes CRC cell growth [32]. Zhang’s research discovered that FBXO22 specifically targets the nucleus to ubiquitinate PTEN, thereby promoting its degradation through the proteasome. This occurs through the promotion of ubiquitination at nuclear PTEN Lys221 residues, ultimately leading to increased proliferation of CRC cells [33].

Interestingly, in contrast to the role of the E3 ubiquitin ligases described above, some other E3 ubiquitin ligases act as oncogenic factors in CRC. For instance, TRIM16, a RING-finger E3 ubiquitin ligase, directly binds to and ubiquitinates Snail, a crucial transcription factor in epithelial-mesenchymal transition. TRIM16 promotes the degradation of Snail and inhibits CRC metastasis [34]. According to a study, CHIP, a member of the U-box family, has been found to inhibit NF-κB signaling in CRC cells by facilitating the ubiquitination and degradation of the NF-κB complex subunit P65. This inhibition leads to a decrease in CRC cell migration and invasion. The study also found that the expression of CHIP is downregulated in CRC at advanced stages. Additionally, subcutaneous tumorigenesis experiments conducted in nude mice showed that tumor growth was slower in the CHIP overexpression group [35]. The HECT domain in the HERC3 protein mediates K48 ubiquitination of RPL23A, with Lys78, Lys89 and Lys123 being the primary sites of HERC3-mediated ubiquitination. Through the promotion of RPL23A ubiquitination-mediated degradation, HERC3 regulates the c-myc signaling pathway and inhibits the proliferation of CRC [36]. In the same year, the laboratory also discovered that the HECT structural domain of HERC3 also induces the degradation of EIF5A2 through ubiquitination at Lys47, Lys67, Lys85, and Lys121. This process inhibits CRC metastasis and regulates the EMT process via EIF5A2/TGF-/Smad2/3 signaling. Another member of the HECT family, SMURF2, inhibits CRC cell proliferation by promoting ubiquitination and degradation of ChREBP, a metabolic switch between glycolysis and oxidative phosphorylation, thereby reducing glycolytic metabolism [37, 38]. MAT IIα, an important enzyme in methionine metabolism, has been found to be associated with uncontrolled cell proliferation in cancer, specifically in CRC. Studies have shown that the levels of Cullin family CUL3 and MAT IIα proteins are negatively correlated in CRC tissues. Further research has shown that CUL3 inhibits CRC cell proliferation by targeting MAT IIα for degradation [39]. The development of colon carcinogenesis is significantly influenced by E3 ubiquitin ligases, which impact protein ubiquitination and proteasomal degradation.

Several inhibitors targeting E3 ligases have shown potential in influencing CRC progression. Recent research has highlighted the role of FBW7, an E3 ligase, as a crucial tumor suppressor in degrading various oncogenes, such as Myc, c-Jun, cyclin E, mTOR, Notch-1 and Mcl-1. In the context of CRC, trametinib has been found to enhance FBW7-mediated ubiquitin‒proteasomal degradation of Mcl-1, ultimately promoting TRAIL-induced apoptosis [40]. Moreover, high expression of USP7, a deubiquitinase, has been observed in CRC and is associated with a poor prognosis. USP7 enhances Wnt signaling activity by stabilizing the β-catenin protein, which contributes to tumor growth. Tao et al. identified an inhibitor called P5091, which promotes ubiquitination and proteasomal degradation of the β-catenin protein, exerting oncogenic effects in CRC [41]. Another USP7 inhibitor, PTL, reported by Li et al. in 2020, demonstrates the potential to inhibit CRC progression by targeting USP7 activity and Wnt/β-catenin protein signaling [42]. The E3 ligase SCFFBXL1 targets the tumor suppressor MEN1 for ubiquitination and degradation. However, two inhibitors, DT1 and SZLP204-1, have been discovered to block SCFFBXL1, resulting in the accumulation of MEN1 protein. This accumulation reduces the proliferation and migration of CRC cells, indicating the potential of SCFFBXL1 inhibitors as CRC treatment [43]. Recent research has underscored the significance of E3 ligases and deubiquitinating enzymes in CRC development and progression. Moreover, autophagy, in addition to the well-known UPS, has been identified as a crucial protein degradation pathway in CRC.

**Fig. 2:**
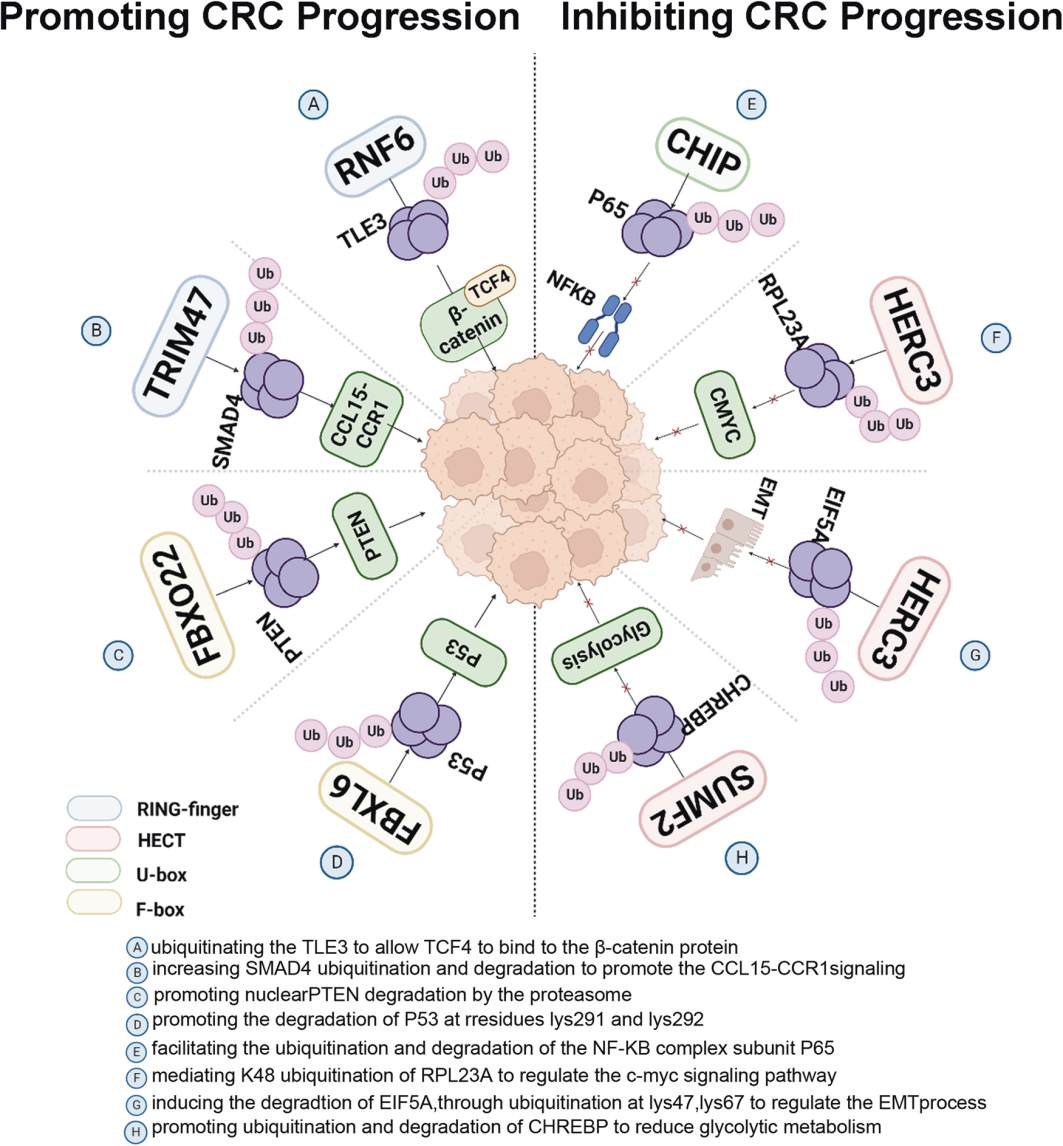
E3 ubiquitin ligases play different roles in CRC evolution. E3 ubiquitin ligases play a critical role in the initiation and advancement of colorectal carcinogenesis by tagging substrate proteins for ubiquitination and subsequent degradation of key proteins within the signaling pathway. Through their regulation of ubiquitination and degradation processes, E3 ubiquitin ligases can act as either promoters or inhibitors of tumorigenesis.

In summary, E3 ubiquitin ligases play a significant role in the development of CRC. While some E3 ubiquitin ligases act as tumor suppressors by promoting the degradation of oncogenic factors, others act as oncogenic factors themselves by inhibiting the degradation of tumor suppressors or promoting the degradation of metabolic switches (Fig. 2). The regulation of protein ubiquitination and proteasomal degradation by E3 ubiquitin ligases is crucial in the development of colon carcinogenesis.

#### Mechanism of action of deubiquitinating enzymes in CRC

Ubiquitination is a reversible process that is regulated by deubiquitinating enzymes. These enzymes remove ubiquitin from target proteins, preventing their degradation and reversing functional changes caused by ubiquitination [8]. The deubiquitinating enzymes are categorized into five main families: the ubiquitin carboxy-terminal hydrolase (UCH) family, the ubiquitin-specific protease (USP) family, the Otubain (OTU) family, the Josephin structural domain protein family and the JAB1/MPN/Mov34 metalloenzymes (JAMM) family [44] (Table 1). Numerous studies have indicated that the USP family plays a crucial role in the development of CRC by impeding protein degradation through deubiquitination. The tumor suppressor FBW7 targets oncoproteins, including Myc, for ubiquitination and is known to mutate in several human cancers. In their research, Nikita Popov et al. discovered that USP28 obstructed the interaction between the F-box protein FBW7 and Myc by binding to the FBW7α chain [45]. This resulted in the stabilization of Myc in the nucleus and promoted the proliferation of CRC cells. Inhibition of USP28 can interfere with the function of Myc in tumors. According to a study, USP9X works against the progression of CRC by regulating the protein stability of FBW7. This is achieved by reducing Myc expression and antagonizing the ubiquitination of FBW7 [46]. USP4, on the other hand, is a member of the USP family and contains three representative structural domains: the N-terminal DUSP (11-122), the UBL structural domain (142-226 and 483-571), and the C-terminal USP catalytic structural domain (302-923). The UBL structural domain of USP4 has been found to have a positive effect on WNT/β-catenin signaling. This is achieved by regulating the protein stability of β-catenin through interaction with the USP4 protein. This promotes the migration and invasion of CRC cells [47]. However, USP44, another member of the USP family, exerts an opposing effect by increasing the protein level of Axin1 through deubiquitination, thereby inactivating the Wnt/β-linked protein pathway and consequently inhibiting proliferation while enhancing apoptosis in CRC cells [48]. Sun and colleagues identified another USP family member, USP11, that stabilizes PPP1CA by deubiquitination. This positively regulates the ERK/MAPK signaling pathway in a PPP1CA-dependent manner, ultimately promoting malignant progression of CRC [49]. Conversely, the proteins OUTB1 and OUTB2 are involved in promoting CRC. OUTB1 prevents the degradation of β-catenin protein through the UPP pathway, leading to increased stability of β-catenin and promoting CRC [50]. Meanwhile, OTUB2 is upregulated in CRC and blocks the interaction between pyruvate kinase 2 (PKM2) and ubiquitin ligase E3, which inhibits PKM2 ubiquitination and degradation. This leads to an increase in PKM2 activity and glycolysis, giving CRC cells a metabolic advantage and promoting their proliferation, migration, and resistance to chemotherapy [51]. Limited research has been conducted on the involvement of the UCHL family in CRC. A single study in 2012 revealed that increased expression of UCHL1 is linked to lymph node metastasis in CRC. This study also found that UCHL1 suppresses the degradation of β-catenin protein through deubiquitination activity, leading to the activation of the β-catenin protein/TCF pathway, and ultimately promoting CRC progression [52].

**Table 1:** The effect of aberrant protein degradation mediated by E3 ubiquitin ligases and DUBs on CRC.

Ubiquitin enzymes	Gene name	Target protein	Relative signal pathway	Promote/Inhibit	Impact on CRC	PMID
E2	UBE2T	P53	P53	Promote cancer	Promote the proliferation, migration, and invasion of CRC cells	32736946
	UBE2J1	RPS3	NFKB	Inhibit cancer	Inhibit the proliferation, migration, and invasion of CRC cells	36567344
E3	FBXL6	P53	P53	Promote cancer	Promote the proliferation of CRC cells	33568778
	FBXO22	PTEN	PTEN	Promote cancer	Promote the proliferation of CRC cells	32249768
	RNF6	TLE3	WNT	Promote cancer	Promote the proliferation of CRC cells	29374067
	FBXL20	E-cadherin	EMT	Promote cancer	Promote migration, and invasion of CRC cells	24932313
	HERC3	EIF5A2		Inhibit cancer	Inhibit the migration and invasion of CRC cells	35064108
	TRIM65	ARHGAP35		Promote cancer	Promote migration and invasion of CRC cells	31332286
	HERC3	RPL23A	c-Myc	Inhibit cancer	Inhibit the proliferation of CRC cells	35637966
	SMURF2	ChREBP	Metabolism	Inhibit cancer	Inhibit the proliferation of CRC cells	31409643
	CUL3	MAT IIα		Inhibit cancer	Inhibit the proliferation of CRC cells	27213918
	SMURF2	SIRT1	Epigenetics	Inhibit cancer	Inhibit the proliferation of CRC cells	32361710
	RBBP6	IκBα	NFKB	Promote cancer	Promote migration, and invasion of CRC cells	31685801
	MAGI3	c-Myc		Inhibit cancer	Inhibit CRC cell growth, promote of CRC cell apoptosis, and chemical sensitivity to fluoropyrimidine chemotherapy.	35864508
	CHIP	P65		Inhibit cancer	Promote tumor growth and decrease tumor cell migration and invasion.	24302614
	FBX8	GSTP1		Inhibit cancer	Inhibit the proliferation, migration, and invasion of CRC cells	31024008
	RNF2	IRF4	Interferon	Promote cancer	Promote the proliferation, migration, and invasion of CRC cells	34670117
	TRIM21	MICALL2		Inhibit cancer	Inhibit migration, and invasion of CRC cells	36307841
DUB	PSMD14	ALK2	BMP6	Promote cancer	Promote the proliferation of CRC cells	31685442
	USP28	c-myc		Promote cancer	Promote the proliferation of CRC cells	24960159
	OTUB1	β-catenin	WNT	Promote cancer	Promote the proliferation ability and chemical resistance of CRC cells	35354355
	USP44	Axin1		Inhibit cancer	Inhibit the proliferation of CRC cells and promoting their apoptosis	32285989
	USP4	β-catenin		Promote cancer	Promote the migration, and invasion of CRC cells	26189775
	UCHL1	β-catenin		Promote cancer	Promote the proliferation of CRC cells	22641175
	OTUB2	PKM2	Metabolism	Promote cancer	Promote the proliferation of CRC cells	34671086
	USP20	SOX4	EMT	Promote cancer	Promote the transfer of CRC	35405623
	USP11	PPP1CA	ERK-MAPK	Inhibit cancer	Promoted the growth and transfer of CRC	31521612

Indeed, the deubiquitinating enzyme activity of DUB counteracts the function of E3 ubiquitinase, which would otherwise lead to protein degradation through the UPS system. This activity inhibits the proliferation, migration and invasion ability of CRC cells by increasing the stability of oncogenic factors. However, it can also promote colon carcinogenesis by stabilizing pro-carcinogenic factors.

## Autophagy–lysosome system

### Introducing autophagy

The autophagy‒lysosome system is primarily responsible for the degradation of long-lived proteins, insoluble protein aggregates and damaged organelles. This process is categorized into three pathways: macroautophagy, microautophagy and chaperone-mediated autophagy (CMA) [53]. Macroautophagy involves the formation of autophagosomes, which are double-membrane vesicles that sequester cytoplasmic constituents and then fuse with lysosomes [54]. Microautophagy is a process in mammals in which materials are engulfed through direct invagination of the lysosomal membrane [55]. Unlike macroautophagy and microautophagy, CMA does not require membrane-bound intermediates. Instead, chaperones directly recognize substrate proteins and deliver them from the cytosol to lysosomes [56]. Recent studies have demonstrated that CMA targets only soluble proteins and not intact organelles due to its specificity [57].

Autophagy is regulated by signals related to metabolism and growth. The key upstream regulator of autophagy is threonine serine kinase (ULK1), which is mainly modulated by the PI3K/Akt/mTORC1 and AMPK/mTORC1 pathways. mTORC1, a crucial player in nutrient sensing and autophagy regulation, is inhibited by nutrient stress or rapamycin treatment to activate autophagy [58]. Moreover, recent studies have demonstrated that mTORC2 functions as a versatile regulator of autophagy. It can either inhibit autophagy through the AKT pathway or promote autophagy via the PKC pathway. In addition, mTORC2 is responsible for controlling basal autophagy and is involved in regulating RTK signaling and maintaining the viability of CRC cells [59–61].

The autophagy‒lysosome system was initially discovered as a means of maintaining intracellular protein and organelle quality and as a means of recycling nutrients. However, it was later found that autophagy is also an adaptive process that helps maintain cellular homeostasis during times of nutrient deprivation or oxidative stress. This is achieved via the breakdown and recycling of proteins and organelles to ensure normal cellular function. The purpose of this review is to summarize the various roles of autophagy in regulating different aspects of CRC progression and development. Although autophagy has been linked to CRC, its exact role in the disease is still a matter of debate.

### The impact of the autophagy‒lysosome system on diverse aspects of CRC

#### The impact of autophagy on CRC cell features

Autophagy has a significant impact on CRC cell proliferation, as it influences various cellular processes. First, autophagy regulates the cell cycle by degrading proteins involved in cell cycle control, such as estrogen receptor beta (ERβ)-mediated autophagic degradation of cell cycle protein D1, leading to cell cycle arrest and a decrease in cell proliferation [62]. Additionally, autophagy regulates oncogenic signaling pathways, such as the Wnt signaling pathway, by promoting the selective degradation of Dishevelled (Dvl), a key component of this pathway, which can negatively regulate Wnt signaling and CRC progression [63]. Furthermore, the loss of SNX10, a protein involved in maintaining lysosomal homeostasis, can impair autophagosome and lysosome fusion and degradation of the nonreceptor tyrosine kinase SRC. The accumulation of SRC and the activation of downstream SRC-STAT1 and SRC-CTNNB10 signaling pathways can contribute to the initiation and progression of CRC [64]. Moreover, autophagy interacts with CMA to maintain cellular homeostasis. When macroautophagy is blocked, CMA can assist in the degradation of mutant p53 [65, 66]. Conversely, when CMA is impaired, cells can rely more on macroautophagy for the degradation of long-lived proteins [67]. Interestingly, studies have shown that SNX10 deficiency inhibits macroautophagy while increasing CMA activity, leading to reduced substrate protein p21Cip1/WAF1 degradation. This promotes CRC cell survival and proliferation and activates mTORC1, a key regulator of cell growth and metabolism, thereby facilitating tumor progression [68]. Overall, autophagy and its interactions with other cellular processes play a complex role in CRC cell proliferation, contributing to the regulation of cell cycle progression, oncogenic signaling pathways, and lysosomal homeostasis. Further research is needed to fully understand the mechanisms underlying these processes and their potential as targets for therapeutic intervention in CRC.

Autophagy also plays a role in the metastasis of CRC cells, which is a major factor contributing to poor prognosis. There are several mechanisms through which autophagy influences CRC metastasis. For instance, RSL1D1 (ribosomal L1 domain containing 1) inhibits autophagy through the RSL1D1/RAN/STAT3 regulatory axis, promoting CRC cell proliferation, invasion and metastasis [69]. Additionally, the E3 ubiquitin ligase TRAF6 catalyzes the K63-linked polyubiquitylation of LC3B and recognizes CTNNB1 for selective autophagic degradation, thereby inhibiting epithelial-mesenchymal transition (EMT) and CRC metastasis [70]. Furthermore, noncoding RNAs (ncRNAs) can regulate autophagy and affect CRC cell metastasis. For example, miR-338-5p inhibits autophagy by targeting PIK3C3, thereby promoting CRC migration, invasion, and metastasis [71]. Cyclic RNA ubiquitin-associated protein 2 enhances autophagy and promotes CRC progression and metastasis through miR-582-5p/FOXO1 signaling [72]. Tissues from CRC patients with metastasis are often enriched with *Fusobacterium nucleatum (F. nucleatum)*. *F. nucleatum* infection has been found to increase CRC cell viability, activate autophagy, and promote CRC metastasis by regulating the expression of various autophagy-related proteins, such as CARD3, LC3-II, Beclin1, Vimentin and P62 [73]. Furthermore, certain autophagy genes can affect CRC invasion in an autophagy-independent manner. High expression of ATG9B in tumors significantly increases the risk of CRC metastasis and poor prognosis. Mechanistically, ATG9B interacts with MYH9 and promotes integrin β1 activation, leading to accelerated focal adhesion (FA) assembly and promoting CRC invasion and metastasis primarily through autophagy-independent mechanisms [74]. These findings indicate the complex involvement of autophagy in CRC metastasis and its impact on various molecular pathways and regulatory factors. Further research is needed to uncover additional mechanisms and potential therapeutic targets related to autophagy and CRC metastasis.

Autophagy and genomic instability are interconnected in cancer. Genomic instability is an enabling characteristic of tumors that leads to abnormal proliferation, genomic alterations and mutations in genes involved in cell division and tumor suppression [75]. Autophagy plays a crucial role in maintaining genomic stability, and disruptions in autophagy can contribute to increased genomic instability and promote carcinogenesis in the early stages of tumors. UVRAG (UV radiation resistance-associated gene) is an important autophagy inducer and tumor suppressor. In CRCs with microsatellite instability, a mutated form of UVRAG called UVRAG^FS^ is present. This mutant form does not inhibit autophagy but instead triggers oncogenic transformation and tumor metastasis by antagonizing the activity of normal UVRAG in autophagy and chromosome stability [76], It also promotes chemosensitivity by directly inhibiting DNA damage repair, leading to increased cell death [77]. The interaction between Beclin 1 and UVRAG plays a role in regulating DNA damage and repair processes, utilizing nonhomologous end joining, and maintains centrosome stability in response to radiation, thus preserving genomic stability [78]. Defects in the ABHD5 gene result in the cleavage of the autophagy gene BECN1 by CASP3. This impairs BECN1-induced autophagic flux, leading to increased genomic instability and promoting carcinogenesis [79]. Overall, these findings highlight the important interplay between autophagy and genomic stability in cancer development. Disruptions in autophagy pathways and associated genes can lead to increased genomic instability, which is a contributing factor in tumor progression and metastasis. Further research is needed to fully understand the mechanisms and potential therapeutic targets involved in this relationship.

#### The impact of autophagy on CRC stemness

Cancer stem cells (CSCs), also referred to as tumor-initiating cells, have strong self-renewal and multilineage differentiation capabilities [80]. In the case of CRC, CSCs are a significant contributor to drug resistance, cancer recurrence and metastasis [81]. Autophagy has been found to contribute to the maintenance of CSC stemness and confer resistance to anticancer therapies. For instance, in CRC, CSCs that are abundant in the tumor exhibit a higher rate of autophagosome formation than cancer cells that are not CSC-rich [82]. Moreover, photodynamic therapy (PDT) induces autophagy, which has an antiapoptotic effect on CRC stem cells, suggesting that inhibiting autophagy may increase the sensitivity of these cells to PDT [82]. On the other hand, activation of autophagy enhances the resistance of CRC cells to oxaliplatin by inducing the aggregation of CD44+ CSCs [83]. The mechanism by which autophagy maintains CSC stemness is not yet fully understood. It has been demonstrated that autophagy can also play a role in inhibiting CSC stemness. For example, QW24, a small molecule inhibitor that targets BMI-24, has been found to significantly suppress the self-renewal of CRC-initiating cells (CICs) in stem-like CRC cell lines by inducing autophagy-lysosome system-mediated degradation of BMI-1, a key regulator for maintaining the self-renewal of CICs. This ultimately leads to inhibition of proliferation and metastasis [84].

#### The impact of autophagy on the immune response in CRC

Autophagy has a significant impact on the immune response in CRC. It regulates immune cell function and cytokine production, thereby influencing various aspects of the immune response [85]. In epithelial cells (IECs), the absence of Stat3 leads to increased mitochondrial autophagy, resulting in the accumulation of iron(II) in lysosomes. This triggers lysosomal membrane permeabilization and the release of proteases into the cytoplasm. This, in turn, enhances MHC class I presentation and CD8 T-cell activation through cross-modification of dendritic cells (DCs) [86]. However, in CRC cells, cytoprotective autophagy plays a tumor-promoting role and contributes to resistance to chemo- and radiotherapy in colitis-associated CRC (CAC) [87]. Autophagy induction mediated by Cathepsin S (Cat S) leads to increased autophagic flux and M2-type polarization of tumor-associated macrophages (TAMs). This ultimately promotes tumor development [88].

Inhibition of autophagy has been shown to have beneficial effects on the immune response in CRC. Coincubation of CRC cells with the autophagy inhibitor chloroquine (CQ) and low concentrations of 5-fluorouracil (5-FU) resulted in increased expression of DC maturation markers, indicating enhanced maturation of antigen-presenting cells. This led to increased expression of perforin and granzyme B in CD8 + T cells and enhanced T-cell response capacity stimulated by DCs [89]. Autophagy also influences the differentiation of a specific class of CD4+ helper T cells called TH9 cells. The transcription factor PU.1 is needed for TH9 cell generation, but p62-dependent selective autophagic degradation of PU.1 inhibits TH9 cell differentiation. Blocking autophagy with CQ enhances TH9 cell differentiation, leading to increased IL-9 secretion. This enhances the anticancer abilities in mouse models of CRC [90]. Overall, autophagy has complex effects on the immune response in CRC. It can modulate immune cell function, antigen presentation, T-cell activation, and differentiation of specific T-cell subsets, all of which contribute to the antitumor immune response. Further research is needed to fully understand the intricacies of autophagy’s role in CRC immunology and explore its potential as a therapeutic target.

#### The impact of autophagy on CRC chemotherapy and resistance

Although the role of autophagy in CRC remains controversial, it is still considered a promising therapeutic target. Many studies have focused on inhibiting or using autophagy inhibitors. One example is the use of pitavastatin to block autophagy, which results in the accumulation of FOXO3a and activation of the PERK-CHOP pathway. Ultimately, this leads to CHOP-mediated apoptosis of cancer cells [91]. Chloroquine and its derivatives have been found to suppress autophagy by impeding the fusion and degradation of autophagosomes and lysosomes. Studies have shown that chloroquine treatment or inhibition of autophagy through downregulation of beclin1 or ATG5 can increase sensitivity to oxaliplatin under normal and hypoxic conditions [92]. However, despite these promising results, clinical trials have not yet demonstrated sufficient efficacy for the use of chloroquine. A new PIK3C3/VPS34 kinase inhibitor called 36-077 has been developed to block autophagy. When combined with 5-fluorouracil (5-FU), it effectively suppresses autophagy and GSK-3β/Wnt/β-catenin signaling, leading to the inhibition of CRC growth. The use of the PIK3C3/VPS34 kinase inhibitor 36-077 may further improve the effectiveness of CRC treatment [93]. Additionally, there are also drugs that can enhance chemotherapy sensitivity by promoting the autophagic degradation of major regulators in anticancer resistance. The FOXM1 nuclear protein is a pivotal regulator of chemoresistance in different types of cancers. Treatment with STL427944, a FOXM1 inhibitor, can lead to the translocation of FOXM1 protein from the nucleus to the cytoplasm, where it can then be degraded by autophagosomes. This process can increase the sensitivity of cancer cells to conventional chemotherapy drugs, including platinum drugs, 5-FU and taxanes [94].

#### The impact of autophagy genes on the prognosis of patients with CRC

Autophagy genes, as autophagy regulators, have different effects on the prognosis of CRC patients with abnormal expression. The loss of LC3B is associated with a poor prognosis [95], while high expression of the autophagy gene SERPINA1 is linked to longer overall survival (OS), recurrence-free survival (RFS) and distant metastasis-free survival (DMFS) in CRC patients [96]. In CRC, a high level of LAMP3 expression is significantly associated with a worse OS [97]. A high level of Beclin 1 expression is linked to a better prognosis in CRC patients [98, 99]. However, it has also been suggested that high levels of Beclin 1 are associated with reduced survival rates in patients treated with 5-FU [100].

As we have discussed, autophagy plays a crucial role in regulating CRC progression by affecting various cellular processes such as the cell cycle, signaling pathways, cancer stemness, immune differentiation and escape, CRC treatment response and drug resistance. However, the exact mechanism behind the switch from anticancer to protumor effects of autophagy is still unclear. Furthermore, there is substantial evidence to suggest that the autophagy‒lysosome system and the UPS are closely interconnected in CRC. In this article, we explore the interconnectedness between these two pathways in CRC.

## The interplay between the UPS and the autophagy‒lysosome pathway

The ubiquitin-proteasome system and the autophagy-lysosome system are pivotal pathways in maintaining protein homeostasis, and these two systems are intricately intertwined: the UPS and autophagy exhibit distinct roles in diverse ailments and are profoundly dysregulated in pathological conditions. Hence, delving into both strategies holds significant merit [101].

**Fig. 3:**
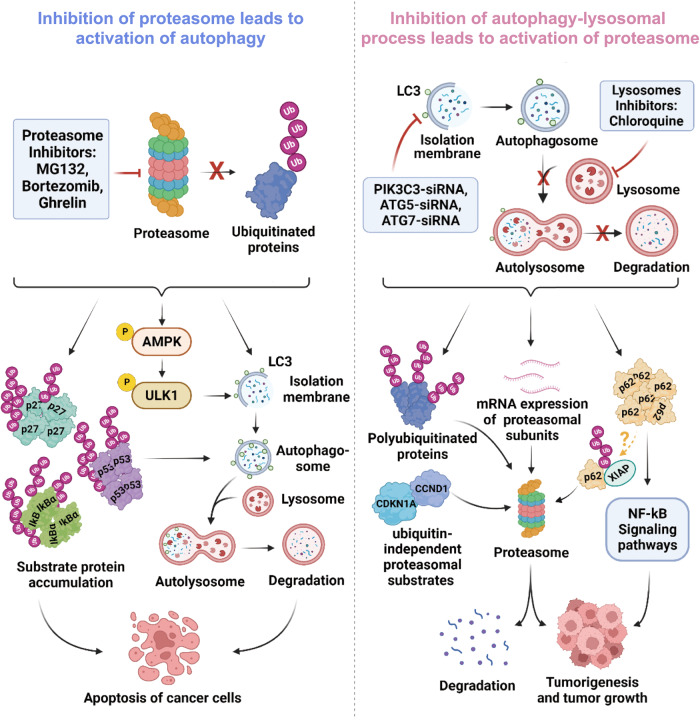
Compensatory relationship between the ubiquitin‒proteasome pathway and the autolysosome pathway. Proteasome inhibitors can impede the degradation and accumulation of ubiquitinated proteins while also inducing autophagy through direct and indirect mechanisms, ultimately culminating in the apoptosis of cancer cells. In addition, inhibition of the autolysosome pathway through RNA interference and lysosomal inhibitor treatment can enhance proteasome activity and substrate protein degradation while also promoting cancer cell genesis and growth via p62 accumulation and its E3 ubiquitin ligase.

Initially, it is crucial to note the intimate correlation between these two methodologies. Ubiquitination serves as a critical mechanism by which selective autophagy achieves precise substrate specificity [102]. The predominant destiny of proteins labeled with ubiquitin is to be recognized by the receptor and subsequently transported to the 26 S proteasome for degradation. However, previous studies have revealed that selective autophagy can also participate in ubiquitinated protein degradation [103, 104]. The linkage between these two pathways is established by ubiquitin molecules, which act on distinct lysine residues to generate diverse ubiquitin chain structures and thus exert different functions (Fig. 3). Autophagy receptors, such as p62/SQSTM1 (p62), optineurin (OPTN), NBR1 and NDP52 have been identified for targeting ubiquitinated proteins to lysosomes [105–108]. Recent studies have indeed discovered PTX80, a new compound that specifically targets the autophagy receptor p62/SQSTM1. This compound binds to p62, resulting in a reduction of soluble p62 levels and the formation of insoluble p62 aggregates. Consequently, polyubiquitylated proteins are unable to colocalize with p62, leading to proteotoxic stress and activation of the unfolded protein response. Ultimately, this cascade of events triggers apoptotic cell death. These findings suggest that targeting autophagy receptors could hold promise as a potential strategy for cancer treatment [109]. Additionally, the ubiquitin‒proteasome pathway, a type of posttranslational modification, can regulate important proteins in the autophagy pathway [110–112]. Certain studies have demonstrated that E2 enzymes, E3 enzymes, and deubiquitinating enzymes can affect the progression of CRC by modifying specific proteins involved in autophagy activation or inhibition. For instance, the aforementioned UBE2V1 suppresses the expression of autophagy genes within CRC [22]. RNF186, an E3 ubiquitin ligase, that is predominantly expressed in the colon and small intestine, is essential for maintaining basal autophagy. RNF186 binds and ubiquitinates EPHB2, which recruits MAP1LC3B to promote autophagy and preserve intestinal homeostasis [113]. ULK1 is a protein located downstream in the autophagy pathway, which is regulated by mTORC1. When mTORC1 is active, it impedes ULK1 activity, thereby preventing the initiation of autophagy. However, when mTORC1 is inhibited, ULK1 is activated, leading to the initiation of autophagy. Recent studies have revealed that ULK1 can also be downregulated through ubiquitination by the E3 ligase NEDD4L. Despite the active transcription of ULK1 mRNA, newly synthesized ULK1 activity is inhibited by mTOR upon reactivation of mTOR-dependent protein synthesis. However, basal ULK1 levels are rapidly restored to prepare cells for potential autophagy stimulation [114]. The deubiquitinating enzyme USP11 has been shown to play a role in autophagy regulation in CRC. Specifically, USP11 facilitates autophagy via the AMPK/Akt/mTOR signaling pathway, leading to the activation of ULK1 and the initiation of autophagy. Additionally, USP11 has been shown to enhance the resistance of CRC cells to 5-fluorouracil treatment, suggesting that it may play a role in drug resistance in CRC. Further research is needed to fully understand the role of USP11 in autophagy and CRC progression [115]. The deubiquitinating enzyme USP5 plays a vital role in the survival of colorectal cancer (CRC) cells, bolstering tumor growth and conferring resistance to chemotherapeutic agents [116]. In both drosophila and mammalian cells, downregulation of Leon/USP5 yields a significant upsurge in autophagosome formation and autophagic flux. This suggests that Leon/USP5 potentially serves as a crucial mediator, bridging the gap between the ubiquitin-proteasome system (UPS) and autophagy pathways [117].

The relationship between these two protein degradation systems is primarily demonstrated through mutual compensation. In the direction of ubiquitin‒proteasome regulation of autophagy, a commonly studied phenomenon is the activation of autophagy upon inhibition of the proteasome by compounds. For instance, MG132, a proteasome inhibitor, has been shown to not only increase the expression of LC3I and LC3II, the activate autophagy but also suppress CRC cell proliferation in vitro [118]. Ghrelin directly inhibits the 20 S proteasome, leading to the accumulation of apoptosis-related proteins p27 and IkBα and promoting apoptosis of CRC cells, while the accumulation of p53 protein activates autophagy [119]. Additionally, bortezomib has been found to induce cytoprotective autophagy in CRC cells through the AMPK-ULk1 signaling cascade [120]. Furthermore, recent studies have demonstrated that the autophagic lysosomal pathway, known as proteaphagy, is responsible for the degradation of the proteasome. Inhibition of the proteasome using bortezomib (BTZ) or MG132 leads to an augmented presence of proteasomal subunits within the lysosome, and this process is independent of chaperone-mediated autophagy (CMA). However, in cells deficient in autophagy-related genes ATG5 and ATG7, the delivery of inactivated proteasomes to the autophagy pathway is only partially impeded [121]. This phenomenon has been observed in various organisms, including Arabidopsis thaliana, yeast, and mammalian cells. Perpetual activation of proteaphagy may occur in certain cancers or when chemoresistance manifests in patients [122]. Such insights provide a novel avenue for identifying therapeutic targets in CRC.

Inhibition of the autophagy–lysosomal process triggers proteasome activation. Knockdown of autophagy-related proteins by RNA interference or lysosomal inhibition by chloroquine leads to elevated mRNA levels of proteasome subunits PSMA5, PSMA7, PSMB1, PSMB5 and PSMD4, thereby enhancing proteasome activity. The increased accumulation of polyubiquitinated proteins and ubiquitin-independent substrates CCND1 and CDKN1A was concomitant with the inhibition of autophagy-induced proteasome activity, which was needed for their degradation [123]. Inhibition of autophagy results in the accumulation of p62, an adaptor protein that delays the delivery of ubiquitinated proteins to the proteasome, thereby inhibiting their removal. The persistent expression of p62 due to defective autophagy can alter the regulation of NF-κB and gene expression, ultimately promoting tumorigenesis [124, 125] XIAP, an E3 ubiquitin ligase of p62, was found to suppress the expression of p62 through ubiquitin‒proteasomal degradation. However, in that study, the authors did not determine whether these p62 were attributed to dysregulation of autophagy. Moreover, XIAP can promote the growth, proliferation, and colony formation of CRC cells in vitro by causing p62 depletion [126]. Combined with p62’s role in other types of cancer, its significance is much more extensive [127–129]. However, maintaining physiological levels of p62 may pose a challenge in cancer treatment. While ubiquitination and autophagy can compensate for each other to maintain protein balance within the body, their signaling mechanisms in CRC require further investigation.

## The relationship between protein degradation and apoptosis

During ubiquitination and autophagy, apoptosis is always an inevitable event in the pathway of cell death [119, 130]. Apoptosis and these two pathways of degraded proteins are also inextricably linked. First, there are apoptosis-related proteins that are regulated by ubiquitination and can be either degraded by ubiquitin ligases or protected from degradation by deubiquitinating enzymes. This regulation directly affects the apoptosis of cancer cells and regulates the degree of cellular resistance to chemotherapeutic drugs. For instance, the E3 ubiquitin ligase FBW7 targets the ubiquitinated antiapoptotic protein Mcl-1 in a glycogen synthase kinase 3 phosphorylation-dependent manner to control apoptosis [131]. Mutations in FBW7 lead to impaired degradation of Mcl-1, which in turn increases resistance to multi-kinase inhibitors of the RAS/RAF/MEK/ERK signaling pathway (including regorafenib and sorafenib) in the clinic [132]. USP1 has been identified as a deubiquitinating enzyme of the antiapoptotic protein Mcl-1, and ML323, a small molecule inhibitor of USP1, has been shown to increase the sensitivity of CRC cells to DNA-targeted chemotherapeutic agents [133]. Alternatively, autophagy, also known as type II programmed cell death, and apoptosis can often collaborate in regulating cell survival and death [91, 134, 135]. Although apoptosis is not a major player in degrading proteins, caspases also cleave proteins to induce their degradation. For instance, T300A, a common variant of ATG16L1, significantly enhances ATG16L1 cleavage by caspase 3 [136]. Chemotherapy-induced apoptosis inhibits autophagy during the execution phase after cytochrome c release, in part through caspase 8-mediated cleavage of Beclin 1 at the D133 and D146 loci [137]. Caspases have tumorigenic capacity as well as invasive and metastatic potential in CRC [138]. Moreover, advanced CRC patients with low levels of caspase-3 respond well to 5-FU chemotherapy [139]. Although it is unclear whether caspase cleavage proteins play a role, direct targeting of caspases or activation of their downstream effector proteins could be a better approach for CRC treatment than the traditional induction of caspase enzymes by compounds/drugs that cause apoptosis.

## Prospects and future directions

In addition to E3 ligases, the UPS has also been the target of targeted protein degradation (TPD) therapies in the last two decades [140]. There are two main related therapeutic approaches. One approach is through proteolysis targeting chimeras (PROTACs), which have shown promise in CRC. PROTACs are stapled polypeptides, such as xStAx-VHL, that can degrade specific proteins such as β-catenin protein in CRC cells. They target and continuously degrade the target protein, thereby inhibiting the Wnt/β-catenin signaling pathway. This approach has demonstrated significant anticancer effects in tumor organoids from CRC patients [141]. Another approach involves the use of molecular glues. For instance, in metastatic CRC, NCT02 acts as a molecular colloid that induces the ubiquitination and proteasomal degradation of cyclin K (CCNK) and its partner CDK12, leading to apoptosis of cancer cells [142]. These two mechanisms, PROTACs and molecular glue, have contributed to the rapid development of TPD therapies. Several related drugs, such as ARV-110 and ARV471 for prostate and breast cancer, respectively, have entered the second stage of clinical trials [143]. Drugs targeting IKZF2, IKZF1/3 and GSPT1 using molecular glues are also in the clinical research stage [144]. This highlights the significant application potential and market value of TPD therapy. Despite these advancements, there is still room for further development in the field of TPD therapy. For PROTACs, expanding the range of targetable E3 ubiquitin ligases is an important area of exploration. Additionally, there is a need for rational design and development of molecular glue drug candidates, moving away from accidental discoveries. Overall, targeted protein degradation has emerged as a promising approach in the treatment of cancers, including CRC. Continuing research and development efforts hold the potential for expanding the application and impact of TPD in the future.

In conclusion, understanding the interaction and molecular mechanisms of protein degradation pathways is crucial for comprehensively analyzing the pathogenesis of CRC (CRC). This review has highlighted the dual role of the ubiquitin‒proteasome system (UPS) and the autophagy–lysosomal pathway in CRC, emphasizing their impact on CRC cell features, signaling pathway modulation, immune cell differentiation, stemness maintenance, treatment response and drug resistance. While these pathways can have opposing functions in CRC initiation and progression, they can complement each other to maintain protein homeostasis. The intricate interplay between the UPS and the autophagy–lysosomal pathway requires further exploration, and future research should focus on understanding the shared roles of multiple protein degradation pathways in CRC therapy. Combination therapies that target both the UPS and the autophagy–lysosome pathway hold promise for overcoming drug resistance and improving patient outcomes. Another area of research with potential is the development of new diagnostic tools that can identify patients who are most likely to benefit from targeted therapies. By identifying specific biomarkers associated with the dysregulation of protein degradation pathways in CRC, clinicians may be able to tailor treatment plans to individual patients and improve overall treatment outcomes. Overall, the study of protein degradation pathways in CRC is a rapidly evolving field with immense potential for improving our understanding of this disease and developing new and more effective treatments for patients.
